# Organizational determinants of psychological safety and occupational stress in healthcare: a phenomenological study in a tertiary health system

**DOI:** 10.1186/s12913-026-15215-5

**Published:** 2026-08-13

**Authors:** Ibrahim Alghamdi, Shadi Ashour

**Affiliations:** Department of Medicine, King Faisal Specialist Hospital & Research Centre, Madinah, Saudi Arabia

**Keywords:** Psychological safety, Occupational stress, Healthcare workforce, Health services research, Patient safety, Organizational culture, Health systems

## Abstract

**Background:**

Modern healthcare ecosystems are shaped by rising service demands, structural workforce shortages, and heightened organizational complexity. While individual-level burnout has been widely evaluated, the underlying organizational determinants that systematically influence psychological safety and occupational stress require deeper investigation. This study explores how healthcare structures, leadership execution, and workload governance shape these dynamics within a transforming tertiary health system.

**Methods:**

A qualitative, interpretive hermeneutic phenomenological design was utilized to examine the lived experiences of a multidisciplinary cohort. Twenty healthcare professionals (*N* = 20) across fifteen distinct clinical departments were recruited via purposive sampling. Data were generated through concurrent qualitative streams consisting of anonymous open-ended surveys (*n* = 12) and in-depth semi-structured interviews (*n* = 8). Thematic analysis was executed using Braun and Clarke’s six-phase framework. Qualitative rigor was maintained via data triangulation, independent parallel coding, and database auditing.

**Results:**

The thematic synthesis identified six core interconnected themes: (1) systemic workload velocity and staffing imbalances, (2) structural suppression of psychological safety and communication barriers, (3) perceived organizational injustice in resource allocation, (4) reactive and isolated managerial leadership styles, (5) compounding impacts on workforce performance and clinical safety, and (6) the critical mandate for system-level occupational interventions. The findings demonstrate that vertical hierarchies, inequitable workload distribution, and punitive management responses are primary drivers of occupational stress and directly inhibit employees’ willingness to voice clinical concerns.

**Conclusions:**

Psychological safety and occupational stress are primarily determined by systemic organizational structures rather than individual resilience profiles. Mitigating healthcare workforce strain and protecting patient care quality requires moving away from individual-focused interventions and toward system-level reforms. Key recommendations include implementing transparent workload allocation models, establishing inclusive leadership frameworks, and institutionalizing psychologically safe reporting channels.

**Supplementary Information:**

The online version contains supplementary material available at https://doi.org/10.1186/s12913-026-15215-5.

## Key Messages

• **Structural Primacy:** Psychological safety is shaped by organizational structures and leadership behavior rather than individual resilience profiles.

• **Stress Drivers:** Systemic workload velocity and acute staffing imbalances serve as the primary institutional drivers of occupational stress.

• **Voice Suppression:** Vertical communication hierarchies and punitive leadership styles directly suppress employee voice and degrade institutional trust.

• **Equity Deficits:** Perceived organizational injustice regarding resource allocation undermines workplace culture and workforce morale.

• **Systemic Mandate:** Structural, system-level interventions are essential for building a sustainable and resilient healthcare workforce.

**Supplementary Information:**

The online version contains supplementary material available at https://doi.org/10.1186/s12913-026-15215-5.

## Introduction

Healthcare systems globally are experiencing unprecedented operational strain driven by shifting demographic demands, systemic workforce shortages, and accelerating institutional complexity [1, 2]. These challenges reshape the workplace environment for healthcare professionals, with direct implications for workforce stability, institutional efficiency, and patient outcomes [1, 3]. To address these challenges, health services research must look beyond individual coping strategies and examine the underlying organizational structures that influence employee wellbeing and communication culture [3, 4].

The clinical workplace is characterized by high cognitive loads, emotional strain, and continuous exposure to critical incidents [1, 5]. These conditions frequently contribute to occupational stress, burnout, and workforce attrition in healthcare systems worldwide [3, 4, 6]. Shift preferences and severe operational environments can accelerate negative psychological outcomes among local frontline care cohorts [6]. While the World Health Organization classifies burnout as an occupational phenomenon resulting from unmanaged workplace stress [7], healthcare leadership often responds with individual-focused interventions like mindfulness training [8, 9]. This approach can inadvertently shift responsibility onto the employee while leaving systemic issues—such as excessive workload, poor resource governance, and unsupportive management practices—largely unaddressed [4, 8, 10].

Psychological safety is an essential component of high-performing healthcare teams [11]. Defined as the shared belief that a team environment is safe for interpersonal risk-taking, psychological safety allows staff to speak up, admit errors, ask questions, and propose new ideas without fear of negative professional consequences [11, 12]. In high-stakes clinical settings, open communication is critical; when horizontal communication is suppressed, errors can go unrecorded, and opportunities for systemic learning may be lost [11, 13]. However, fostering psychological safety is often challenged by traditional medical hierarchies and punitive management approaches [12, 13].

Leadership behavior serves as a primary driver of psychological safety and workforce stress [14, 15]. Supportive, inclusive leaders who seek out feedback and prioritize team wellbeing help reduce occupational strain and increase staff engagement [4, 14]. Conversely, leadership marked by low transparency, poor communication, or inequitable resource allocation can worsen stress and contribute to a culture of silence [13, 16].

These dynamics are particularly visible in healthcare systems undergoing rapid, macro-structural modernization. For instance, Saudi Arabia’s healthcare system is currently undergoing a comprehensive national transformation [6, 17]. The implementation of accelerated transformation programs introduces new operational frameworks, structural expectations, and shifts in institutional culture [17]. While these changes aim to improve service efficiency, they can also introduce acute workplace stress and challenge established communication patterns [6, 17]. Traditional clinical environments often maintain rigid, vertical command structures that can suppress psychological safety [11, 13]. Fostering inclusive leadership and equitable resource management is therefore essential to help buffer high operational demands [4, 14].

The Job Demands–Resources (JD-R) model offers a valuable framework for understanding these dynamics [4, 18]. This model posits that occupational stress develops when high job demands (e.g., severe workload, emotional strain) are not balanced by adequate job resources (e.g., leadership support, operational autonomy, psychological safety) [4, 18]. Within this framework, psychological safety serves as a critical organizational resource that helps buffer the impact of high workload demands [11, 19]. Similarly, High Reliability Organization (HRO) theory emphasizes that in complex, high-risk environments, open communication and proactive leadership are vital for identifying system vulnerabilities before they lead to adverse events [20, 21].

While the importance of these factors is increasingly recognized, there remains a need for qualitative research examining how they are experienced across multidisciplinary healthcare teams [11, 22]. Qualitative approaches are uniquely suited to capture the complex, real-world dynamics of clinical workplaces and provide a deeper understanding of the factors influencing workforce wellbeing and patient safety [23, 24]. High levels of occupational stress and low psychological safety are associated with reduced staff retention, increased medical errors, and compromised quality of care [3, 25]. Qualitative methods help uncover these underlying systemic drivers [23].

The explicit aim of this study is to explore how organizational structures, leadership execution, and workload governance determine psychological safety and occupational stress among a multidisciplinary cohort within a tertiary healthcare system, providing structural insights that are highly relevant for healthcare leaders and policymakers.

## Methods

### Study design

A qualitative, interpretive hermeneutic phenomenological design was employed to explore healthcare professionals’ lived experiences of occupational stress and psychological safety within organizational contexts [24, 26, 27]. Grounded in the interpretive tradition, this design acknowledges that individuals’ lived experiences cannot be divorced from their socio-cultural and organizational environments [24, 27]. This approach is particularly appropriate for investigating complex workplace dynamics—such as leadership influence, organizational culture, and implicit institutional norms—that are not easily captured using quantitative methods [23]. By emphasizing lived experience, hermeneutic phenomenology enables the exploration of both emotional and cognitive dimensions of occupational stress, as well as the ways in which existing organizational structures facilitate or inhibit psychological safety [24].

**Reflexive Methodological Justification:** Blending anonymous qualitative surveys with face-to-face semi-structured interviews was a deliberate, conceptually reflexive choice. Because this inquiry explicitly evaluates psychological safety deficits and punitive hierarchical behaviors within an institution, relying solely on visible, face-to-face interviews risked introducing severe selection and response biases. Providing an anonymous, secure, text-based data stream was ethically and practically imperative to capture the unvarnished, authentic lived experiences of vulnerable or fearful frontline staff members who would have otherwise declined participation due to retaliation concerns. This multi-stream approach successfully cross-verified the systemic nature of institutional silence while preserving participant safety.

### Setting and participants

The study was conducted within a large tertiary healthcare institution in Saudi Arabia, which provides specialized medical services across a wide range of clinical specialties [6, 17]. This setting provides an appropriate environment for investigating how complex organizational structures affect a multidisciplinary workforce.

A total of twenty (*N* = 20) healthcare professionals from fifteen distinct clinical departments were recruited using purposive sampling to ensure relevant experience across different operational roles [28]. The cohort consisted of 11 male and 9 female professionals, with tenures ranging from 6 months to more than 10 years of institutional experience. Eligible participants included frontline clinicians and supervisory staff with direct patient care responsibilities and a minimum of six months of experience in the institution [28]. Clinical support staff were operationally defined as non-prescribing, patient-adjacent professionals, including case managers, clinical unit coordinators, and social workers. To ensure analytical transparency, the detailed demographic and institutional characteristics of the participants across their respective data collection streams are presented in Table 1.

**Table 1:** Demographic and institutional characteristics of study participants (*N* = 20). This table outlines the cohort characteristics distributed across sex, tenure, qualitative data collection streams, and clinical specialties

Characteristic	Category	n
Sex	Male	11
	Female	9
Professional experience	6 months–5 years	8
	>5 years	12
Data collection method	Anonymous online survey	12
	Semi-structured interview	8
Survey participant departments (*n* = 12)	Internal Medicine	2
	Emergency Medicine	2
	General Surgery	1
	Adult Critical Care	1
	Radiology (MRI)	1
	Respiratory Therapy	1
	Psychiatry	1
	Social Services	1
	Oncology	1
	Laboratory Services	1
Interview participant roles (*n* = 8)	Medical-Surgical Registered Nurse	2
	Emergency Room Registered Nurse	1
	Pediatrician	1
	Occupational Therapist	1
	Physical Therapist	1
	Clinical Dietitian	1
	Paramedical Staff	1

### Data collection

Data collection utilized two concurrent, complementary qualitative streams to support data triangulation and provide multiple avenues for participant expression:

### Anonymous surveys

Open-ended, anonymous electronic surveys were completed by twelve (*n* = 12) participants from various acute and support departments using a secure internal platform [11]. This approach allowed staff to share perspectives on sensitive organizational topics—such as leadership performance or perceived workplace inequities—without risk to their professional standing, which is an important consideration in hierarchical healthcare environments [13, 16]. The open-ended questions focused on everyday workplace stressors, resource equity, and departmental communication.

### Semi-structured interviews

To gain deeper insight into these lived experiences, detailed semi-structured interviews were conducted with a separate subset of eight (*n* = 8) multidisciplinary professionals [28]. Interviews were held in private offices to ensure confidentiality and encourage candid discussion [28]. The interview guide was developed based on existing literature and feedback from the research team to address key areas including workplace stress, leadership behavior, communication patterns, and psychological safety [3, 4, 11, 29]. Informed consent was obtained both in writing and orally prior to session initialization.

Example prompts included:

*“Describe an incident where you consciously chose not to speak up in your department.”*

*“How do current operational resource allocations affect your day-to-day stress levels?”*

*“What changes would improve psychological safety in your department?”*

Interviews lasted between 30 and 60 minutes and were audio-recorded with the explicit consent of the participants. This approach follows established standards for qualitative health services research [23].

Sample size determination was guided by meaning and thematic saturation [23]. Saturation was monitored concurrently during iterative data collection and analysis cycles [23]. Thematic saturation was recognized during the 16th informant check cycle, at which point further data generation produced no new codes, structural categories, or substantive modifications to the thematic framework [23]. Four additional data reviews were completed to confirm the stability of the final code architecture across both the survey and interview cohorts.

### Data analysis

The transcripts were analyzed using thematic analysis, following the six-phase framework established by Braun and Clarke [30, 31]. The research team first immersed themselves in the data through repeated reading of the transcripts to ensure complete familiarization. Initial codes were generated through an inductive, line-by-line coding process conducted independently by the researchers. These codes were then grouped into broader conceptual categories mapping onto organizational determinants to search for overarching themes.

The emerging themes were reviewed iteratively by cross-examining the coding structures against the raw transcript excerpts to ensure contextual accuracy. Themes were then formally defined, named, and integrated into a structured narrative. Peer debriefing and cross-coder validation were utilized throughout the analysis to enhance credibility and minimize individual interpretive bias, maintaining a clear audit trail from raw transcripts to the final thematic output.

**Theoretical Convergence Note:** To align this analytical tool with our stated phenomenological orientation, Braun and Clarke’s reflexive thematic analysis framework was executed from an experiential, essentialist standpoint. This orientation focused explicitly on mapping the structural essence and systemic realities of the participants’ workplace environments. By centering on the structural meanings embedded within raw accounts, we effectively bridged the practical data-reduction mechanics of thematic analysis with the deeper philosophical requirements of interpretive hermeneutic phenomenology.

### Reflexivity

The research team maintained an active reflexive practice throughout the study lifecycle, documenting assumptions, professional backgrounds, and potential interpretive biases in a reflexive journal. This ongoing review supported analytical transparency and helped ensure that interpretations remained closely grounded in the participants’ actual narratives.

### Trustworthiness

Several strategies were implemented to safeguard the trustworthiness of the findings. Credibility was supported through data triangulation across the survey and interview streams, allowing for cross-verification of themes. Peer debriefing helped refine the coding architecture and thematic definitions. Transferability was supported by providing detailed descriptions of the institutional setting, operational definitions, and participant characteristics, allowing readers to evaluate the relevance of the findings to other healthcare environments [28]. Dependability and confirmability were maintained through a systematic audit trail consisting of raw transcripts, codebooks, and reflexive notes.

### Ethical considerations

This study was reviewed and approved by the Institutional Review Board of the participating healthcare institution (IRB No. 2,251,643). All participants provided informed consent prior to data collection. Participation was entirely voluntary, and individuals retained the right to withdraw from the study at any point without penalty. All data were fully anonymized during transcription by removing institutional and individual identifiers. Data were stored securely on encrypted drives accessible only to the primary research team, in accordance with the Declaration of Helsinki [32].

## Results

### Overview of findings

Thematic analysis of the multidisciplinary narratives identified six interconnected themes describing the organizational determinants of psychological safety and occupational stress. To enhance credibility, transparency, and trustworthiness, the findings are presented alongside isolated participant quotes appended with unique alphanumeric codes indicating the informant’s professional discipline and operational role tier.

**Table:** 

Theme	Subtheme	Representative Qualitative Extract	Source Code
1. Workload Velocity & Staffing Imbalances	• High Patient-to-Staff Ratios • Depleted Recovery Windows	“We are often expected to manage more patients than is safe, and there is rarely enough time to rest between shifts. It feels like the pressure never ends.”	PN-03 (Frontline Nurse)
2. Suppression of Psychological Safety	• Punitive Hierarchy • Retaliation Vulnerabilities	“Sometimes I notice errors or unsafe practices, but I hesitate to speak up because I worry about being criticized or sidelined by senior leadership.”	PA-09 (Allied Health)
3. Perceived Organizational Injustice	• Inequitable Allocations • Recognition Deficits	“Some staff members consistently get lighter workloads, while others are left with the most challenging patients. It feels unfair and demoralizing.”	PN-06 (Frontline Nurse)
4. Insufficient Managerial Support	• Isolated Leadership • Metric-Driven Focus	“When things get stressful, management is often absent or focused only on numbers rather than understanding our struggles.”	PP-04 (Frontline MD)
5. Impacts on Performance & Safety	• Cognitive Exhaustion • Communication Failures	“When I am overwhelmed or anxious, it affects how I interact with patients and colleagues. Mistakes become more likely, and the quality of care suffers.”	PN-12 (Frontline Nurse)
6. Mandate for System Interventions	• Policy-Driven Safety • Structural Reallocation	“Training on resilience is helpful, but what we really need are changes at the organizational level—fair workload, supportive managers, and a culture where we can speak up safely.”	PN-02 (Frontline Nurse)

### Theme 1: systemic workload velocity and staffing imbalances

Participants consistently identified high workload velocity and chronic staffing imbalances as primary drivers of acute occupational stress [4]. Informants reported that high patient volumes and extended shifts left insufficient recovery time, leading to persistent fatigue [2]. The physical and psychological exhaustion described by participants was not an episodic occurrence but a persistent state of operational saturation. Informants expressed that the constant drive to meet institutional throughput targets without proportional staffing adjustments severely undermined their capability to provide high-quality care.

“We are often expected to manage more patients than is safe, and there is rarely enough time to rest between shifts. It feels like the pressure never ends.”—Participant Nurse [PN-03, Frontline Role]

These demands were frequently worsened by unexpected short-staffing, which forced teams into a continuous crisis-management mode and accelerated emotional exhaustion [2]. The narratives revealed a pattern where systemic vacancies directly increased the operational burden on the remaining frontline staff.

“The staffing shortage means we are constantly double-booked. You cannot provide deep clinical attention when you are structurally forced to rush through every interaction.”—Participant Nurse [PN-07, Frontline Role]

“The sheer velocity of admissions combined with depleted baseline staffing levels creates an operational environment where we are constantly triaging crises rather than executing planned, comprehensive care.”—Participant Physician [PP-01, Leadership Role]

Participants emphasized that prolonged exposure to these high-demand conditions eroded their professional efficacy and depleted their cognitive reserves, demonstrating that individual coping strategies cannot offset systemic understaffing [1, 4].

### Theme 2: structural suppression of psychological safety and communication barriers

A major finding was the perceived lack of psychological safety across several clinical areas [11]. Participants noted a consistent reluctance to report near-misses, process vulnerabilities, or clinical errors due to a fear of criticism or negative professional consequences [13]. The accounts highlighted an active calculation made by frontline staff, who frequently chose silence over interpersonal risk-taking. This hesitation was directly attributed to an unsupportive organizational climate that prioritizes individual blame over systemic error analysis.

“Sometimes I notice errors or unsafe practices, but I hesitate to speak up because I worry about being criticized or sidelined by senior leadership.”—Participant Allied Health [PA-09, Frontline Role]

The willingness to voice concerns was closely tied to immediate leadership behavior and the broader institutional culture [11]. Punitive responses to errors tended to discourage open communication and reinforce a culture of silence [13].

“When I tried to bring up a repeating error in our supply handovers, I was told to ‘just focus on my work’ and stop creating issues. It completely discourages anyone from trying to improve the unit.”—Participant Allied Health [PA-04, Frontline Role]

“If you expose a workflow failure, the institutional habit is to look for a person to fault rather than assessing the structural glitch. It is simply safer to stay silent.”—Participant Clinical Support [PCS-14, Frontline Role]

These narratives highlight how rigid, vertical hierarchies can suppress essential safety communication, increasing systemic vulnerability [13].

### Theme 3: perceived organizational injustice in resource allocation

Participants frequently noted imbalances in workload distribution, professional recognition, and administrative transparency. Perceptions of unequal allocation or inconsistent policy enforcement contributed to frustration and decreased engagement [33]. The sense of organizational injustice was compounded by a perceived lack of transparency regarding how operational duties and shift rotas were assigned. When allocation choices appeared arbitrary or influenced by personal connections, it severely damaged unit morale and fragmented team cohesion.

“Some staff members consistently get lighter workloads, while others are left with the most challenging patients. It feels unfair and demoralizing.”—Participant Nurse [PN-06, Frontline Role]

These perceived inequities undermined trust in leadership and reduced staff willingness to take on duties outside their core roles. Informants emphasized that transparent and predictable operational practices are essential for maintaining morale [33].

“Decisions about shift scheduling and overtime allocation feel completely hidden behind closed doors. There’s no objective formula, which leaves a lot of us feeling heavily penalized.”—Participant Clinical Support [PCS-02, Frontline Role]

“Even when we go completely above and beyond under intense pressure, our efforts are rarely acknowledged or valued. It creates a deep sense of administrative neglect.”—Participant Allied Health [PA-02, Frontline Role]

These findings indicate that organizational injustice functions as a distinct structural stressor that undermines workplace cohesion and trust [33].

### Theme 4: reactive and isolated Managerial leadership styles

Participants identified unsupportive and isolated management practices as key factors driving both workplace stress and low psychological safety [11]. Leaders were often perceived as distant, with interactions focused heavily on operational metrics rather than staff wellbeing [16]. This leadership gap left many frontline clinicians feeling disconnected from the administrative level. Management was often seen as a policing body focused on compliance rather than an supportive resource designed to facilitate safe clinical operations.

“When things get stressful, management is often absent or focused only on numbers rather than understanding our struggles.”—Participant Physician [PP-04, Frontline Role]

The narratives emphasized the need for visible, collaborative leaders who engage directly with frontline challenges and seek staff input during high-pressure periods [15]. A lack of leadership presence often contributed to feelings of isolation among the staff.

“We only see our department head when an audit is failed or a target is missed. There is no proactive touchpoint, which makes every leadership interaction inherently stressful.”—Participant Allied Health [PA-11, Frontline Role]

“We need leaders who are actually present on the floor during operational bottlenecks, not just administrators issuing top-down commands from closed offices.”—Participant Clinical Support [PCS-18, Leadership Role]

These accounts confirm that reactive management styles reinforce communication gaps and undermine the development of a supportive workplace culture [15, 16].

### Theme 5: compounding impacts on workforce performance and clinical safety

Participants described a direct link between high occupational stress, low psychological safety, and compromised patient care [3]. Chronic stress was reported to impair cognitive focus, disrupt communication, and increase the likelihood of clinical errors [3]. The intersections of psychological distress and operational risk were prominent in the participants’ descriptions of their daily work. Clinicians noted that when they operate under chronic anxiety and fatigue, their clinical decision-making capacity is diminished.

“When I am overwhelmed or anxious, it affects how I interact with patients and colleagues. Mistakes become more likely, and the quality of care suffers.”—Participant Nurse [PN-12, Frontline Role]

Informants noted that emotional exhaustion could impair teamwork and reduce the capacity for patient empathy, posing long-term risks to patient care quality—a dynamic heavily linked to employee management and broader safety parameters within acute hospitals [3, 25, 34].

“There are days when you are so textually saturated with administrative paperwork and rapid turnarounds that you miss minor changes in a patient’s lab trend. It terrifies me, but it’s the rhythm we are forced into.”—Participant Physician [PP-03, Frontline Role]

“The emotional exhaustion gets so high that you lose the capacity for clinical empathy. You’re just trying to survive the shift, which is dangerous for patient safety.”—Participant Physician [PP-05, Frontline Role]

These results underscore the close connection between workforce wellbeing and institutional safety outcomes [3].

### Theme 6: the critical mandate for system-level occupational interventions

Participants consistently argued that individual resilience initiatives are insufficient to address the structural root causes of workplace strain [8, 9]. Instead, they emphasized the clear need for system-level organizational interventions [8, 16, 35]. The narratives showed a shared frustration with institutional strategies that focus on individual adaptation rather than structural change. Informants explicitly called for concrete changes to operational policies, staffing mandates, and leadership accountability frameworks.

“Training on resilience is helpful, but what we really need are changes at the organizational level—fair workload, supportive managers, and a culture where we can speak up safely.”—Participant Nurse [PN-02, Frontline Role]

Recommended improvements included establishing transparent staffing models, implementing inclusive leadership training, expanding accessible mental health resources, and developing non-punitive reporting channels [13, 20].

“Mandatory relaxation modules feel like an insult when you don’t have enough physical bodies on the floor to take a lunch break. Fix the ratios first.”—Participant Nurse [PN-09, Frontline Role]

“Sustainable safety won’t come from workshops. It requires operational policies that enforce safe staffing and protect staff who expose systemic gaps.”—Participant Physician [PP-02, Frontline Role]

These insights align with structural frameworks like the Job Demands–Resources model, showing that meaningful improvements require system-level changes rather than short-term interventions [4].

## Discussion

The findings of this study demonstrate that psychological safety and occupational stress among healthcare professionals are primarily shaped by systemic organizational conditions rather than individual characteristics [4, 11]. Factors such as high workload demands, vertical communication hierarchies, perceived organizational injustice, and distant management styles interact to influence staff behavior, morale, and performance [4, 33]. Together, these elements form an interconnected system that directly affects both workforce wellbeing and patient care quality [3].

Using a qualitative design allowed for a detailed examination of participants’ lived experiences, uncovering complex institutional dynamics that are often missed by quantitative metrics [23]. These insights provide a clearer understanding of how structural factors influence communication and stress in everyday clinical practice [24].

**Fig. 1:**
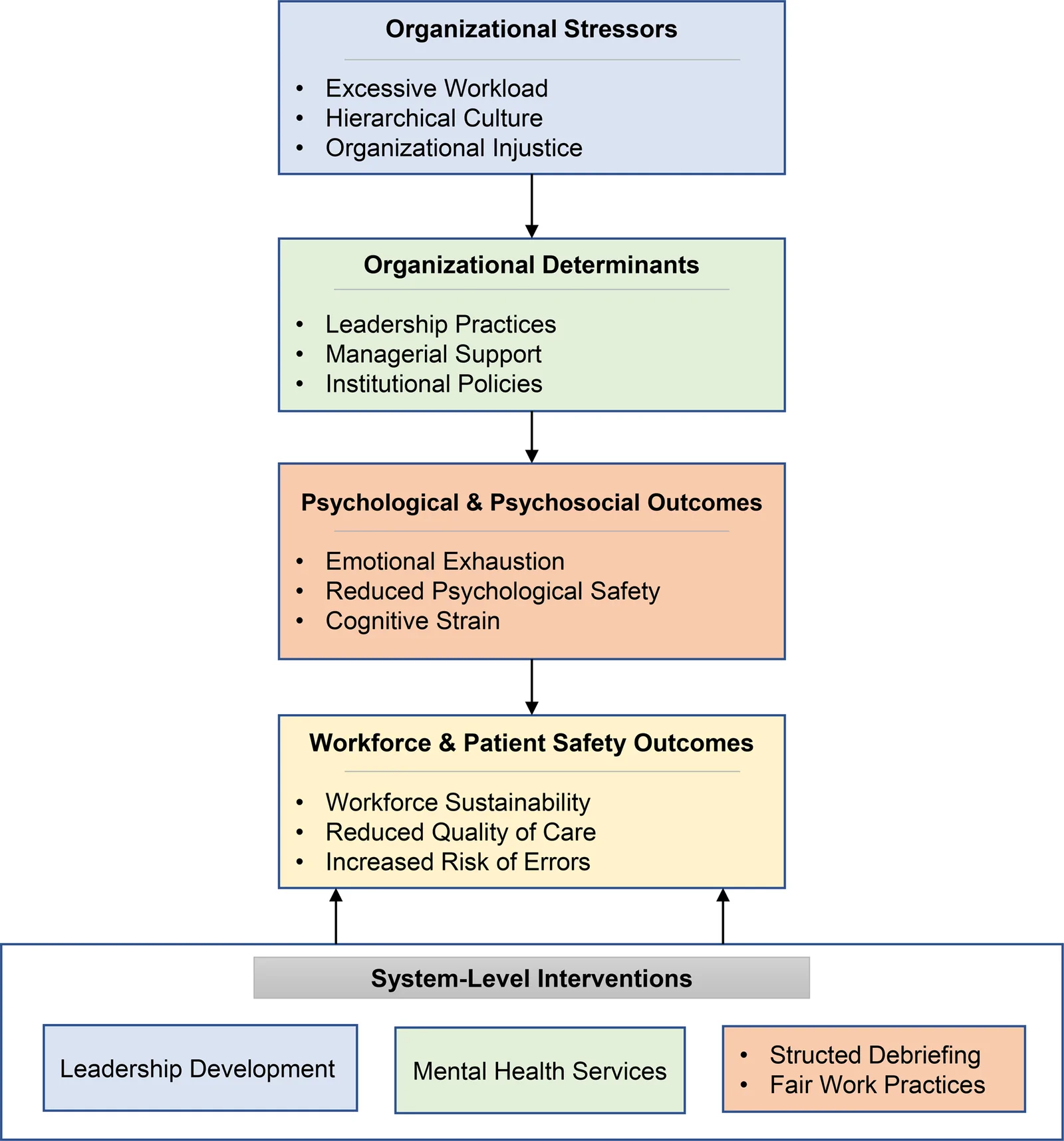
Conceptual framework of systemic healthcare strain and psychosocial cascades. Visual mapping of the directional pathways within transforming tertiary health systems. The framework illustrates how root macro-level organizational stressors dictate localized unit determinants, trigger downstream psychological and clinical safety vulnerabilities, and are mitigated via a restorative system-level intervention loop

Consistent with the Job Demands–Resources (JD-R) framework, this study indicates that occupational stress increases when high job demands (e.g., severe workload velocity, staffing shortages) are not balanced by adequate organizational resources (e.g., leadership support, fair resource allocation, psychological safety) [4, 18]. Crucially, our findings extend this model by positioning psychological safety as a central organizational resource that can help buffer workplace stress and support open communication under high demands [11]. Based on these relationships, we developed a conceptual framework to illustrate how these organizational factors interact to shape workforce wellbeing, staff performance, and patient care outcomes (Fig. 1).

The Structural Architecture of the Conceptual Framework Figure 1 provides a visual map of the explicit, multi-tiered directional pathways through which institutional realities translate into clinical outcomes:

- **Primary Stressor Inflow:** The model establishes that root-level Organizational Stressors (*Excessive Workload, Hierarchical Culture,* and *Organizational Injustice*) act as the initial input variables. These macro-level stressors directly constrain and dictate localized Organizational Determinants, specifically shaping *Leadership Practices, Managerial Support,* and the execution of *Institutional Policies* across clinical units.
- **Psychosocial Cascades:** When these determinants display structural friction—such as reactive management or punitive vertical hierarchies—they trigger negative Psychological & Psychosocial Outcomes. Frontline staff experience this as a direct combination of *Emotional Exhaustion, Reduced Psychological Safety*, and severe *Cognitive Strain*.
- **Downstream System Vulnerabilities:** These psychosocial strains do not remain localized; they manifest downstream as degraded Workforce & Patient Safety Outcomes. The model details how cognitive fragmentation and a culture of silence lead directly to compromised *Workforce Sustainability*, an *Increased Risk of Clinical Errors*, and an ultimately *Reduced Quality of Care*.
- **The Restorative Feedback Loop:** Crucially, the framework introduces an upward pathway via targeted System-Level Interventions (*Leadership Development, Mental Health Services, Structured Debriefing,* and *Fair Work Practices*). Rather than acting as temporary patches on individual symptoms, these interventions are designed as a structural feedback loop that routes backward to directly dismantle, reallocate, and mitigate the foundational *Organizational Stressors*. By treating the system rather than individual coping deficits, long-term workforce equilibrium is achieved.

### Organizational determinants and psychological safety

The lack of psychological safety emerged as a significant structural barrier across several departments [11]. Participants reported a consistent reluctance to speak up due to fear of blame, administrative pushback, or professional marginalization [13]. These findings align with existing research showing that low psychological safety inhibits open communication, collaborative learning, and error reporting [11, 13].

In high-stakes healthcare environments, suppressed horizontal communication can directly impact patient safety [3]. When frontline staff feel unable to raise concerns or question clinical decisions, opportunities for early error detection and systemic improvement are compromised [11]. This underscores the role of psychological safety as an essential organizational resource necessary for maintaining safe clinical practices [11, 12].

Rigid hierarchies were frequently cited as primary obstacles to open communication [13]. Participants noted that vertical command structures and punitive responses to errors discouraged dialogue, particularly among early-career staff [13]. This supports prior research suggesting that traditional, top-down structures can hinder the reporting of near-misses and limit opportunities for process improvement [13]. Conversely, inclusive leadership behaviors that invite input and encourage transparency help build trust and support safer team performance [14].

### Workload pressures and organizational stress

Chronic workload pressure and staffing imbalances were identified as central drivers of occupational stress [4]. Informants described high patient-to-staff ratios, extended shifts, and limited recovery windows as major contributors to chronic physical and emotional exhaustion [2]. These observations are consistent with international evidence linking high job demands to increased burnout and reduced professional efficacy [2, 4].

Our findings also highlight the compounding relationship between high workload and low psychological safety [11]. Under severe time and staffing constraints, healthcare professionals have less capacity for reflective communication and collaborative problem-solving. This suggests that workload governance is an essential organizational responsibility [1, 2]. Ensuring adequate staffing levels and transparent workload distribution is necessary not only to protect workforce wellbeing but also to support patient safety and care quality [1, 3].

### Perceived organizational injustice

Perceptions of organizational injustice served as a distinct source of stress and disengagement [33]. Structural inequities in workload allocation, inconsistent recognition, and a lack of managerial transparency undermined morale and institutional trust [33]. This is consistent with existing health services literature linking organizational injustice to increased emotional exhaustion, lower job satisfaction, and higher turnover intentions [33, 36].

In high-demand environments, maintaining transparency and fairness is critical for sustaining staff engagement [33]. These findings suggest that addressing structural inequities through clear, consistently applied operational policies can help strengthen institutional trust and support psychological safety [11, 33].

### Managerial support and workforce wellbeing

Isolated and reactive management styles were found to contribute directly to both occupational stress and reduced psychological safety [11]. Participants indicated that leadership interactions were frequently limited to monitoring operational targets rather than proactively supporting staff wellbeing [16]. This supports prior research demonstrating that active, supportive leadership is necessary to mitigate burnout and maintain workforce engagement [16].

Effective healthcare leadership requires regular engagement with frontline staff, clear communication, and responsiveness to workplace concerns [15]. Approachable leaders help establish a sense of psychological safety, whereas a lack of visible support can worsen stress and reinforce a culture of silence [13]. This aligns with High Reliability Organization (HRO) principles, which emphasize that leadership presence is vital for fostering communication, continuous learning, and resilience in complex systems [20, 21].

### Implications for patient care

A key contribution of this study is the clear link established between organizational determinants, workforce wellbeing, and clinical safety [3]. The data demonstrate that severe occupational stress and structural suppression of speech do not merely impact individual health; they alter the operational landscape of patient safety. Under conditions of cognitive fatigue induced by high workload velocity, clinician focus narrows, disrupting critical diagnostic cross-checking and multi-professional coordination [3, 25].

Furthermore, when psychological safety is structurally minimized, frontline personnel actively withhold observations regarding medication anomalies, equipment performance failures, or deviations from clinical protocol [11, 13]. Consequently, institutional silence operates as a latent systemic vector for clinical errors. Protecting patient outcomes requires that health institutions treat psychological safety not as an abstract cultural asset, but as an active, clinical safety resource that requires protective infrastructure [11, 20].

### Implications for Health services and Policy

While these findings are context-specific, they are transferable to tertiary healthcare institutions facing similar operational demands, hierarchical structures, and workforce constraints [1, 2]. They provide practical insights into how structural conditions shape communication and wellbeing in high-pressure clinical environments [3].

These insights are highly relevant for healthcare leaders and policymakers, particularly within transforming health systems [17]. Workload governance, leadership development, and institutional culture should be evaluated as core components of health service design [17]. Implementing transparent staffing models and equitable workload distribution may help reduce occupational strain and support staff retention [1, 4].

At the institutional level, leadership programs should focus on cultivating inclusive communication, psychological safety, and active engagement to help build more supportive work environments [14, 15]. Additionally, regular assessments of psychosocial risks can help leaders identify high-strain areas and implement timely interventions [8]. At the system level, embedding workforce wellbeing into national health strategies is essential for building sustainable and resilient health systems during periods of macro-structural transformation [17]. Protecting employee wellbeing and fostering open communication are critical steps for ensuring long-term patient safety and service quality [1, 17].

### Strengths, limitations, and future directions

This study provides qualitative evidence on how organizational factors influence psychological safety and occupational stress across multidisciplinary healthcare teams [11]. It highlights how vertical communication hierarchies and perceived organizational injustice shape staff behavior, and it offers a conceptual framework connecting these structural factors to workforce wellbeing and patient safety [3, 11]. These insights expand existing knowledge by demonstrating how organizational dynamics operate in complex, real-world clinical settings [11].

However, these findings should be considered within the context of the study’s design [28]. As a qualitative, single-center study, the goal was not statistical generalization, but rather an in-depth exploration of lived experiences within a specific institutional environment [28]. While the consistency of the themes across different disciplines suggests these findings reflect common structural patterns, variations in leadership approaches and organizational design may influence how these dynamics manifest in other settings [15, 17].

Additionally, the sample size (*N* = 20), though appropriate for qualitative analysis, represents a relatively small cohort [28]. Nevertheless, the achievement of thematic saturation supports the depth and credibility of the analysis [30]. Finally, self-reported data can be subject to response bias, especially regarding sensitive topics like leadership and institutional culture [28]. To address this, data collection relied on anonymous surveys and confidential interviews to encourage candid feedback [11, 28]. Future research would benefit from multi-center designs and mixed-methods approaches to further evaluate and expand this conceptual framework. In complex healthcare environments, employee silence is rarely accidental—it is structurally produced.

## Conclusion

This study demonstrates that psychological safety, occupational stress, and patient care quality are heavily shaped by systemic organizational determinants. Efforts to improve workforce wellbeing and protect patient safety must move away from individual-focused coping strategies and instead address core structural challenges, such as workload governance, leadership engagement, and communication culture. Fostering psychological safety within clinical teams is essential for improving institutional communication, supporting a resilient workforce, and ensuring high-quality patient care. Without these structural reforms, healthcare systems risk perpetuating environments marked by chronic stress, staff disengagement, and increased safety vulnerabilities.

## Electronic supplementary material

Below is the link to the electronic supplementary material.

Supplementary Material 1

## Data Availability

The qualitative data generated and analyzed during this study are not publicly available due to confidentiality restrictions. Data are available from the corresponding author on reasonable request.

## Abbreviations

DEM: Department of Emergency Medicine
ER: Emergency Room
HRO: High Reliability Organization
IRB: Institutional Review Board
JD-R: Job Demands–Resources model
MRI: Magnetic Resonance Imaging
OT: Occupational Therapy
PT: Physical Therapy
