# Supplementary material for: Organizational determinants of psychological safety and occupational stress in healthcare: a phenomenological study in a tertiary health system
Source: BMC Health Serv Res. 2026 Aug 13;26:1577. doi: 10.1186/s12913-026-15215-5 (PMC13757178; doi:10.1186/s12913-026-15215-5)
Supplement: Supplementary file 1 [file 12913_2026_15215_MOESM1_ESM.docx]

**Semi-Structured Interview Guide**

**Section 1: General Experience**

- Can you describe a typical workday and the challenges you face in your role?
- How would you describe the overall atmosphere and culture in your workplace?

**Section 2: Psychological Health and Safety**

- What does “psychological safety” mean to you in your work environment?
- Have you experienced situations where you felt unsafe expressing your opinions or concerns? Can you describe them?
- What factors in your workplace contribute to stress or psychological strain?

**Section 3: Support and Coping**

- What kind of support do you receive from supervisors, colleagues, or the organization?
- How do you cope with work-related stress or psychological challenges?

**Section 4: Impact and Consequences**

- How does workplace stress or psychological safety affect your job performance, satisfaction, or well-being?
- In your opinion, how does it affect patient care?

**Section 5: Suggestions for Improvement**

- What changes or interventions do you think could improve psychological health and safety in your workplace?
- Are there practices from other organizations or departments that you think could be adopted here?
